# Seroprevalence and burden of hepatitis E viral infection among pregnant women in central Nigeria attending antenatal clinic at a Federal Medical Centre in Central Nigeria

**DOI:** 10.3389/fmed.2022.888218

**Published:** 2022-09-02

**Authors:** Philomena Ehi Airiohuodion, Anh Wartel, Andrew B. Yako, Peter Asaga Mac

**Affiliations:** ^1^Faculty of Medicine, Centre for Medicine, and Society, University of Freiburg, Freiburg, Germany; ^2^Special Programme for Research & Training in Tropical Diseases (TDR), World Health Organization, Geneva, Switzerland; ^3^International Vaccine Research Institute, Seoul, South Korea; ^4^Department of Zoology, Nasarawa State University, Keffi, Nigeria; ^5^Institute of Virology, Universitätsklinikum Freiburg, Freiburg, Germany

**Keywords:** HEV, fetomaternal outcome, IgG, Nigeria, fulminant hepatitis

## Abstract

**Introduction:**

HEV infection may be life threatening in pregnant women and has been linked with 20–30% mortality, especially in the third trimester of pregnancy. HEV infection leads to elevated levels of preterm labour and other immunological parameters. It is vertically transmitted and could lead to poor feto-maternal outcomes. especially in fulminating viral hepatitis where both the mother and foetus could be lost. There is currently no known treatment or vaccine for HEV. There is therefore a need to study HEV seroprevalence and burden among vulnerable groups, such as pregnant women and their newborns in Nigeria, where maternal mortality is highly significant.

**Methods:**

A total of 200 samples were collected from pregnant women attending antenatal clinic at Federal Medical Centre (FMC) Keffi, in central Nigeria, of which (156/200) samples were from HIV-negative pregnant women and (44/200) were from HIV-positive pregnant women, using a simple random sampling method.

**Results:**

In total, 200 pregnant women [78.0% (156/200) HIV-negative pregnant women and 22.0% (44/200) HIV-positive pregnant women] were recruited for this study. The ages of the pregnant women ranged from 15-49 years, with a mean age of 26.4 years (± 6.23). The overall HEV IgG seropositivity in the study population was 31.5% (63/200); 95% CI (30-33).

**Conclusion:**

This study highlighted an unexpectedly high seroprevalence of HEV and poor feto-maternal outcomes in pregnant women residing in a rural and urban setting of central Nigeria. The study showed that the inherently high HEV seropositivity and poor feto-maternal outcomes may not be attributed to HEV viral hepatitis only but may be a combination of extrinsic and intrinsic factors.

## Introduction

Hepatitis E is an infectious disease of the liver caused by the hepatitis E virus (HEV). In countries with limited access to essential water, sanitation, hygiene, and health services or in areas experiencing humanitarian crises, hepatitis E is common (1). HEV is transmitted mainly by the faecal-oral route, primarily through contaminated water. The infection is usually self-limiting and resolves within 2–6 weeks. Occasionally, a serious disease known as fulminant hepatitis (acute liver failure) develops, which can be fatal (1–4). The World Health Organization (WHO) reports that approximately one-third of the world’s population live in areas where HEV is endemic (1, 5, 6). At least 63 countries have reported HEV infections; approximately half of these have reported large outbreaks (5, 7). There are approximately 20 million HEV infections, 3.3 million symptomatic cases of hepatitis E, and 60,000 deaths worldwide annually (2). In addition to hepatic manifestations, extrahepatic manifestations have been reported with HEV infection, including pancreatitis, neurological symptoms, hematological disorders, glomerulonephritis, and mixed cryoglobulinemia (2, 3).

HEV primarily affects young adults and is generally mild; however, the mortality rate is significantly higher for pregnant women, particularly in cases where the infection progresses to fulminant hepatitis, especially in the second and third trimesters, putting pregnant women at increased risk of acute liver failure, foetal loss, and most times death (8–11). The case-fatality ratio can be as high as 20-25% in the last trimester of pregnancy (1).

Several studies from developing countries, including Nigeria, indicate that HEV infection is highly prevalent in pregnancy, and up to 30%-100% of pregnant women develop fulminant hepatitis (1, 4, 9, 10, 12–15). In Africa, particularly in resource-limited countries, disease caused by HEV infections is a major public health problem (10, 16–18). There have been several reports of HEV outbreaks in Chad, January 2022 (1, 7, 19).

In Nigeria, there is a lack of knowledge and awareness about HEV (9, 10, 12–15, 20–26). A study among rural dwellers pregnant women attending antenatal clinics [ANC] revealed a prevalence of 0.4% lower than the general population prevalence of 4% (16). The majority of pregnant women with jaundice and febrile illness who were HEV seropositive exhibited symptoms in their third trimester, and the investigation revealed high HEV antibody levels (9). Another study conducted in Plateau state, Northern Nigeria, showed a pooled prevalence of 42.6% among pregnant participants (20).

Currently, there are limited data available about hepatitis E-related infections among pregnant women in Nigeria. It is therefore imperative to conduct more research to fully understand the magnitude, burden, seroprevalence and foeto-maternal outcomes of the disease among pregnant women to initiate control measures.

## Methods

### Study area

**FIGURE 1:**
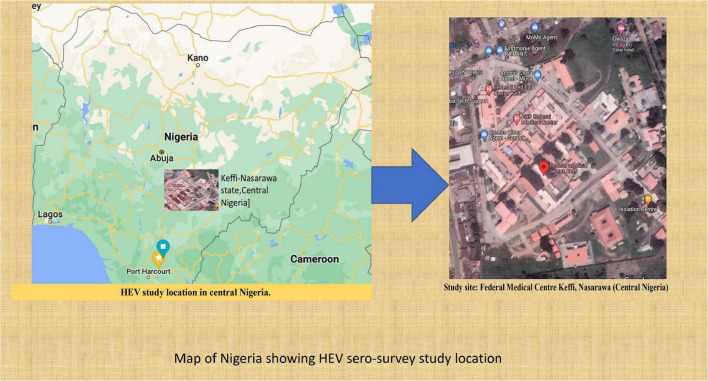
Showing the HEV study site in Central Nigeria.

This study was conducted from December 2019 to August 2020 at the Federal Medical Center Keffi in central Nigeria. Keffi town is a diverse community in Nasarawa state in north central Nigeria. It is located in close proximity to Nigeria’s capital, Abuja. As a result of its strategic location, this area has a high influx of people from other contiguous areas of the country, contributing to its heterogeneity (Figure 1).

### Study design and site

A cross-sectional study was conducted on 200 pregnant women at the Federal Medical Center (FMC) in Keffi, North Central Nigeria. It has one of the largest antenatal clinics (ANCs) and antiretroviral unit attendance per day from many states of Nigeria, including the Abuja FCT. This facility is among Nigeria’s largest hospitals, serving approximately 15-20 million people each year. The total population of the region is over 25 million. Forty-five percent of the population live in urban areas, 40% in rural areas, and 15% in slums or informal settlements (9). The average annual temperature in these areas range from 21°C to 27°C, while in the interior lowlands, temperature are generally above 27°C. The mean annual precipitation is 1,165.0 mm (5, 6, 16). It rains throughout the year in most parts of central Nigeria, with most rainfall occurring between April and October and minimal rainfall occurring between November and March. The main occupation of the people in this region is farming.

### Study population

The study population included pregnant women (HIV-negative) who enrolled for antenatal care and HIV-positive pregnant women who were receiving treatment at the antiretroviral (people living with HIV/AIDS) units of the Federal Medical Centre, Keffi, between December 2019 and November 2020. This centre was purposefully selected to reflect the diversity (in terms of different cultures, religions, ethnicities, topographical and vegetation features, and different human activities) of the geographical regions. The inclusion criteria were pregnant women within an age range of 15-49 years who agreed to participate in the study and signed the consent form, while the exclusion criteria were pregnant participants who were already undergoing treatment for hepatitis E virus infection, those who refused to sign the consent form and seriously ill pregnant patients who were hospitalized.

### Screening of study participants

A structured questionnaire was used to obtain information that included questions on demographics, medical history, vital signs and symptoms, clinical evaluation, data on hospitalization, and a summary form. All study subjects were screened for hepatitis-related symptoms (fever, fatigue, loss of appetite, nausea, vomiting, abdominal pain, jaundice, and dark urine). Detailed protocol information was made available and fully explained to the participants in English and their respective local languages before enrolment. The study participants signed an informed consent form after enrollment. Participants who could not read and write were asked to verbally consent and then to thumb print indicating that they were willing to participate in the study.

A total of 200 samples were collected from the HIV-negative pregnant women (156 samples) and HIV-positive pregnant women (44 samples) employing a simple random sampling method. The sample size calculation (based on a 15% expected proportion of hepatitis E viral infections in a total population of five hundred thousand patients with a confidence interval of 95% and a p value of 0.05) (27) showed a minimum sample size of 196 serum samples, which we increased to 200 samples to be able to analyse subgroups according to sociodemographic characteristics.

Venous blood was collected (5 mL) from pregnant participants by the principal investigator and his assistants. This was done throughout the 12-hour shift. The serum was extracted and thereafter shipped on dry ice to the Institute of Virology, Freiburg, Germany. The serum samples were stored at −20°C in preparation for laboratory analysis for HEV infection.

### Laboratory analysis

All serum samples were tested using hepatitis E serological markers (anti-HEV IgG) (recomLine IgG, Mikrogen GmBH, Germany) according to the manufacturer’s instructions (28). Positive samples were confirmed using recomScan test strip analysis software (recomScan computer software; Mikrogen GmbH) (28). A liver function test (LFT) was further conducted on serum samples of pregnant women who tested positive for HEV IgG biomarkers for alanine aminotransferase (ALT) and bilirubin liver enzymes by a laboratory technician located in the hospital haematological laboratory. The method used was a modified LFT protocol of the Universitatsklinikum Molecular Diagnostic Center.

### Statistical test

Statistical analysis was performed using SPSS version 28. Descriptive statistics were employed for the analysis of results and 95% confidence intervals [CI] to identify the sociodemographic and behavioral characteristics of the study population. The results are presented in tables and figures. A regression analsysis and relative risk performed to examine the relationship between the demographic variables and hepatitis E virus infections(HEV). The results were deemed statistically significant at a p value ≤ 0.05.

### Ethical considerations

Ethical approval was obtained from the Institutional Review Board (IRB) of Federal Medical Centre, Keffi [No. KF/REC/02/21] and Uniklinikum ethical committee, University of Freiburg [No. 140/19].

## Results

### Sociodemographic characteristics of hepatitis E virus among the study population

**FIGURE 2:**
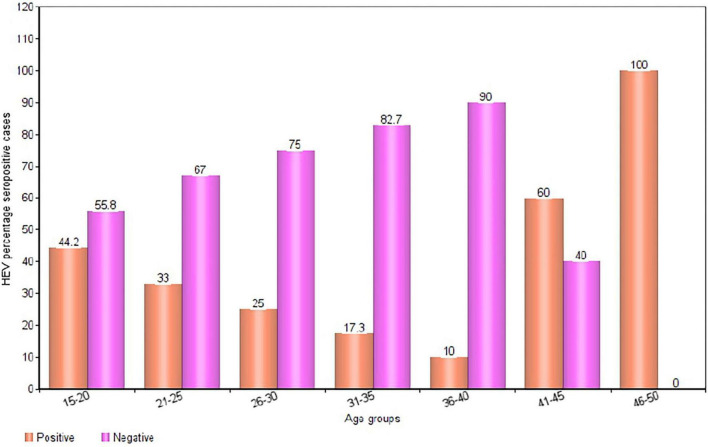
Bar chart showing the age-specific seropositivity of HEV in pregnant women.

**FIGURE 3:**
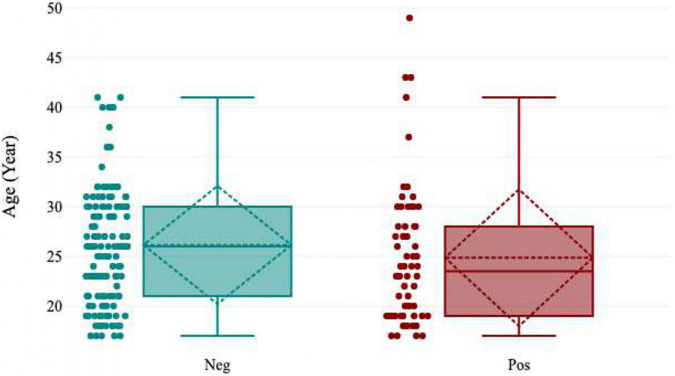
Boxplot showing the age-specific seropositivity of HEV in pregnant women.

In total, 200 pregnant women 78.0% (156/200) HIV-negative pregnant women and 22.0% (44/200) HIV-positive pregnant women] were recruited for this study. The ages of the pregnant women ranged from 15-49 years, with a mean age of 26.4 years (± 6.23). The overall HEV IgG seropositivity in the study population was [31.5% (63/200); 95% CI (30-33)]. The results of a subgroup analysis revealed a markedly high seroprevalence of HEV in the age groups 46-50 years [100% (1/1); 95% CI (90-109)] and 41-45 years [60.0% (3/5); 95% CI (56-64)]. The lowest percentage HEV IgG seropositivity was observed in the 36-40 years age group [10.0% (1/9); 95% CI (7-13)] (Figures 2, 3).

### Sociodemographic characteristics of study participants and HEV seroprevalence

#### Marital status

In the present study, the IgG seroprevalence of HEV in single pregnant women [39.3% (11/2); 0R = 1.5; 95% CI (37-41)] was slightly higher than that in married [31.1% (50/161); OR = 0.9; 95% CI (30-32)] and divorced pregnant women [18.2% (2/11); OR = 0.5; 95% CI (15-21)]. The odds of contracting HEV for single pregnant women were 1.5 times the odds of contracting HEV for pregnant women groups (Table 1).

**TABLE 1:** Sociodemographic characteristics of study participants and HEV seroprevalence.

Marital status	Positive	Negative	Total (*N*%)	95% CI	Odd ratio	*p* value
**Status**						
Married	50 (31.1%)	111 (68.9%)	161 (100%)	30–32	0.9	0.42
Single	11 (39.3%)	17 (60.7%)	28 (100%)	37–41	1.5	
Divorced	2 (18.2%)	9 (81.8%)	11 (100%)	15–21	0.5	
Total (*N*%)	63 (31.5%)	137 (68.5%)	200 (100%)	30–33		
**Source of drinking water**						
Stream	33 (34.0%)	64 (66.0%)	97 (100%)	33–35	1.2	0.68
Borehole	23 (28.0%)	59 (72.0%)	82 (100%)	27–29	0.8	
Tap	7 (33.3%)	14 (66.7%)	21 (100%)	31–35	1.1	
Total (*N*%)	63 (31.5%)	137 (68.5%)	200 (100%)			
**Place of domicile**						
Urban	22 (25.9%)	63 (74.1%)	85 (100%)	25–27	1.6	0.14
Rural	41 (35.7%)	74 (64.3%)	115 (100%)	35–37		
Total (*N*%)	63 (31.5%)	137 (68.5%)	200 (100%)	30–33		
**Educational status**						
Primary	20 (33.9%)	39 (66.1%)	59 (100%)	33–35	1.2	0.56
Secondary	32 (32.9%)	65 (67.1%)	97 (100%)	32–34	1.1	
Tertiary	11 (25.0%)	33 (75.0%)	44 (100%)	24–26	0.7	
Total	63 (31.5%)	137 (68.5%)	200 (100%)	30–33		
Information on HEV						
Yes	45 (29.8%)	106 (70.2%)	151 (100%)	23–35	1.2	0.40
No	18 (36.7%)	31 (63.3%)	49 (100%)	30–42		
Total	63 (31.5%)	137 (68.5%)	200 (100%)	30–33		
Occupation						
Housewife	32 (32.3%)	67 (67.7%)	99 (100%)	31–33	1.1	0.33
Trader	12 (44.4%)	15 (55.6%)	27 (100%)	42–46	2.0	
Civil servant	16 (25.0%)	48 (75.0%)	64 (100%)	24–26	0.6	
Student	3 (30.0%)	7 (70.0%)	10 (100%)	27–33	0.9	
Total	63 (31.5%)	137 (68.5%)	200 (100%)	30–33		
**History of blood transfusion**						
Yes	9 (25.7%)	26 (74.3%)	35 (100%)	24–27	1.2	0.41
No	54 (32.7%)	111 (67.3%)	165 (100%)	32–33		
Total (*N*%)	63 (31.5%)	137 (68.5%)	200 (100%)	30–33		
**Pregnancy in trimesters**						
First trimester	8 (17.0%)	39 (83.0%)	47 (100%)	14–20	0.4	0.03*
Second trimester	6 (27.3%)	16 (72.7%)	22 (100%)	12–31	0.8	
Third trimester	49 (37.4%)	82 (62.6%)	131 (100%)	36–39	2.3	
Total	63 (31.5%)	137 (68.5%)	200 (100%)	30–33		
HIV Status						
HIV-positive pregnant women	14 (31.8%)	30 (68.2%)	44 (100%)	30–33	1.0	0.00*
HIV-negative pregnant women	49 (31.4%)	107 (68.6%)	156 (100%)	30–32		
Total (*N*%)	63 (31.5%)	137 (68.5%)	200 (100%)	30–33		

*Significant.

### Source of drinking water

HEV IgG seropositivity was higher among pregnant women whose source of drinking water was stream [34.0% (33/97); OR = 1.2; 95% CI (0.28-0.40)] and tap [33.0% (7/21); OR = 1.1; 95% CI (31-35)] than among those who drank from the borehole [28.0% (23/82); OR = 0.8; 95% CI (27-29)]. The predicted odds of contracting HEV in pregnant women who sourced drinking water from streams were 1.2 times the odds of pregnant women who accessed drinking water from other sources (boreholes and taps) (Table 1).

### Place of domicile

A remarkably higher seropositivity of HEV was observed in pregnant women who resided in urban areas [74.1% (63/85); 95% CI (25-27)] compared to those who dwell in rural areas [64.3% (74/115); 95% CI (35-37); OR = 1.6; *p* = 0.14)] (Table 1). Pregnant women residing in urban areas had a 1.6 times higher risk of contracting HEV than pregnant women residing in rural areas (Table 1).

### Education level

A significant proportion of HEV IgG seropositivity was detected in pregnant women who had primary [33.9% (20/59); OR = 1.2; 95% CI (33-35)] and secondary [32.9% (32/97); OR = 1.1; 95% CI (32-34)] levels of education, while a lower seropositivity was revealed among pregnant women who were university or tertiary school graduates [25.0% (11/44); OR = 0.7; 95% CI (24-26)]. The odds of contracting HEV among pregnant women who attained a primary level of education were 1.2 times the odds of pregnant women who attained other forms of education (Table 1).

### Knowledge or information about HEV

Pregnant women who had no prior knowledge or information about hepatitis E virus had the highest HEV IgG seroprevalence [36.7% (18/49); 95% CI (30-42)] compared to pregnant women who were aware of disease [29.8% (45/151); 95% CI (23-35)]. The risk of contracting HEV was 1.2 times greater for pregnant women with no prior knowledge or information about the disease than for pregnant women who were aware of the viral infection (RR = 1.2; *p* = 0.40) (Table 1).

### Occupation

A considerable seropositivity rate of HEV IgG [44.4% (12/27); OR = 2.0; 95% CI (42-46)] was observed among traders, while a much lower seroprevalence rate was noticed among civil servants [25.0% (16/64); OR = 0.6; 95% CI (24-26)]. In the present study, the predicted odds of contracting HEV among pregnant women who were traders was 2.0 times the odds for other occupations (Table 1).

### History of blood transfusion

There was a significant proportion of [32.7% (54/165); 95% CI (32-33)] HEV seropositivity among pregnant women who had no history of transfusion compared to those [25.7% (9/35); 95% CI (24-27)] who had a history of transfusion. In the present study, pregnant women who had no history of transfusion had a 1.2 times higher risk of contracting HEV than pregnant women who had a history of transfusion (OR = 1.2; *p* = 0.41) (Table 1).

### Gestational age

**FIGURE 4:**
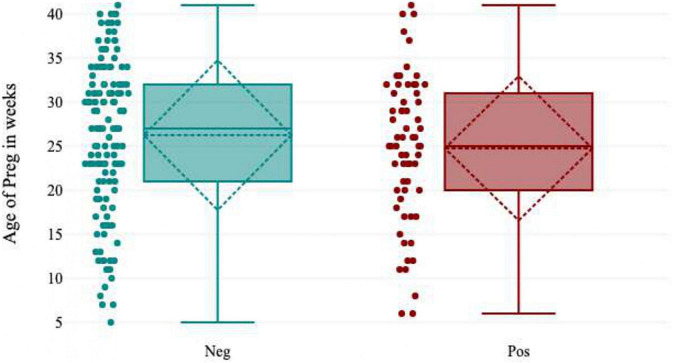
Box-plot showing gestation age of HEV IgG seropositivity distribution.

Pregnant women [37.4% (49/131); OR = 2.3; 95% CI (36-39)] who were in their third trimester showed the highest seropositivity for HEV IgG. There was a much lower IgG seropositivity rate in pregnant women in the first trimester [17.0% (8/47); OR = 0.4; 95% CI (14-20)]. The odds of contracting hepatitis E virus were 2.3 times higher in pregnant women who were in their third trimester of pregnancy than in pregnant women in the first and second trimesters. There was a statistically significant difference between HEV seropositivity and gestational age (*p* = 0.03) (Figure 4 and Table 1).

### HIV status

The seroprevalence of HEV IgG was [31.8% (14/44); 95% CI (30-33)] in HIV-negative pregnant women, and the seropositivity rate detected in HIV-negative pregnant women was [31.4%% (49/156); 95% CI (30-32)]. There was no difference in the two groups in terms of their risk of contracting HEV based on whether they were exposed. The disease was equally likely to occur in each group in the present study. However, there was a statistically significant association between HIV seropositivity and HEV infection (OR = 1.0; *p* = 0.00) (Table 1).

### Fetomaternal outcome of HEV-seropositive pregnant women

**FIGURE 5:**
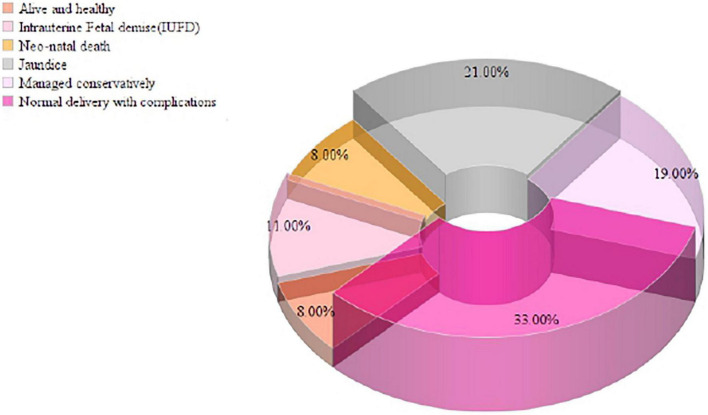
Fetomaternal outcome of HEV-seropositive pregnant women in the study population.

In the present study, 31.5% (63/200) of the pregnant women were positive for HEV IgG biomarkers. Among these, 33.0% (21/63) of the pregnant women participants delivered safely without any medical complications, and 8.0% (5/63) of the women delivered live and healthy babies. There were 11.0% (7/63) intrauterine fetal demise (IUFD), 8.0% (5/63) neonatal death, 21.0% (13/63) of the newborn babies had jaundice, and 19.0% (12/63) of the pregnant women were managed conservatively during delivery and discharged safely. The pregnant women’s serum bilirubin levels ranged from 1-50 mg/dL, with a mean value of 15.83 ± 11.62 mg/dL, and the ALT level ranged from 23-1933 IU/L, with a mean value of 589.2 IU/L. Pregnant women who presented during the third trimester and postpartum had elevated liver enzymes due to late ANC registration and non-compliance with treatment during prenatal periods (Figure 5).

## Discussion

It is well known that infection caused by hepatitis E virus (viral hepatitis) can present unimaginable complications during delivery, including intrauterine fetal death and poor maternal and fetal outcomes among pregnant women (3, 9, 10). To fully understand this event, we conducted a study to determine the seroprevalence, burden and foeto-maternal outcome of HEV among pregnant women in central Nigeria.

An overall HEV IgG seropositivity of 31.5% was observed among the pregnant women participants, compared to the estimated pooled prevalence of 42.6% (9), 28.0% (10) and 13.3% (29) obtained from other pregnant women seropositivity studies conducted in other regions of Nigeria. These differences could be attributed to sample size, and the differences in specificity and sensitivity of HEV detection that resulted from different laboratory diagnostic techniques, including the study location and environment. The physiological state of the women during pregnancy, HEV genomic and viral factors, variant heterogeneity, and hormonal factors also resulted in reduced or altered immune responses to HEV infection. The discrepancies in seropositivity could also be related to socioeconomic status, urban and rural socioinfrastructural differences, such as poor housing, poor sewage and drainage infrastructures, and unhygienic conditions of the environment (flooding and poor sanitary practices) compounded by unwholesome waste disposal practices in various homes.

HEV seropositivity was much higher among older pregnant women who were 46-50 years old(100%) than those who were 41-45 years old (60.0%). The high seropositivity of HEV in the older pregnant women may be explained by the cumulative risk of infection over a subject’s lifetime, whereas the higher IgG antibody concentrations detected in the younger group may reflect the proportion of subjects among all positives who have recently experienced the first HEV infection. Since anti-HEV IgG levels typically decrease with time, those subjects will likely have higher anti-HEV IgG concentrations than those who had the infection in the distant past. There is a greater tendency for acute cases to manifest symptoms in older people.

HEV seroprevalence was higher in single pregnant women (39.3%; OR = 1.5) than in married (31.1%; OR = 0.9) and divorced (18.2%; OR = 0.5) pregnant women. Factors such as intimacy with multiple seropositive partners, low socioeconomic status, poor sanitation, and socio-behavioural practices such as eating and drinking from food vendors (10). Studies have also shown that children are silent carriers of HEV (20), proximity of these single pregnant women or mothers to thier childern might also put them at risk of HEV infection.

In the present study, pregnant women whose drinking water source was stream (34.0%; OR = 1.2) or tap (33.3%; OR = 1.1) showed higher HEV seropositivity than those who drank from boreholes (28.0%; OR = 0.8). The reasons could be social and unhygienic health and waste disposal practices, such as bathing, washing, and feeding of animals, which could lead to contamination of water sources with human and animal excrement. It could also be related to municipal tap water supply channels (pipelines) that are broken or poorly laid, leading to contamination of water sources with sewage and waste matter. Consumption of uncooked meat and drinking of unclean and poorly purified water may also be responsible for the high seropositivity (1, 9–11, 29). Several studies are of the opinion that water sources are significantly associated with HEV infection and outbreaks in most low middle income countries (LMICs) (10, 30, 31). In the current study, HEV seroprevalence in urban areas was higher compared to rural areas, but the result was not statistically significant (74.1%; OR = 1.6; *p* = 0.14). This could be explained by the state of poor sewage infrastructure in urban settings, unhygienic conditions of the environment, compounded and aggravated by sewage handling practices, consumption of undercooked meat (zoonosis) and unsafe and broke water supply pipelines or channels (9, 11, 16).

Our study revealed a markedly seropositive rate of HEV among pregnant women who attained primary (33.9%) and secondary (32.9%) level of education compared to pregnant women who attained tertiary level (25.0%) of education. This may be attributed to a low literacy level, poor hygiene, unwholesome cultural practices, and poor socioeconomic status. Education could be a valid predictor of HEV infection; nonetheless, there is no consensus about this association in other studies (10).

Pregnant women without prior knowledge or information about HEV (36.7%; OR = 1.2; p = 0.40) had a higher seroprevalence of HEV viral hepatitis than pregnant women with prior knowledge and information (29.8%). The reason could be related to a low literacy level, poor hygiene, and unwholesome sanitary and culture practices among the ill-informed pregnant women group.

Civil servants (25.0%) showed a much lower HEV seroprevalence than traders (44.4%), housewives (32.3%) and students (30.0%), which could be attributed to the level of educational attainment among career pregnant women. This could also be related to the use of selective sources of information on HEV, relying on critical thinking attitudes and making more active choices and informed decisions to prevent HEV infection. Best sanitation practices and access to clean or safe water could be the other reasons (9–11, 17, 29–32). Pregnant career women could also have better economic standing (often high-income earners), thereby preferring to eat at home with less exposure to contaminated food and water.

Pregnant women with no history of blood transfusion (32.7%; OR = 1.2) were more likely to contract HEV than pregnant women (25.7%) who had a blood transfusion history, which could be explained by lack of awareness of transfusion-mediated spread (3) and poor sanitary and cultural practices, because majority of the pregnant women came from diverse sociosettings.

The seroprevalence of the hepatitis E virus was highest in pregnant women, especially those in their second (27.3%) and third (37.4%) trimesters, according to this study. It could be related to a declining immune state, as well as pathophysiological changes that occur as pregnancy progresses (10, 31). This could also be attributed to prolonged viral factors, such as viremia viral protein, and elevated viral load due to chronic infection. However, this study did not investigate these viral factors (2, 3, 12).

There was no marked difference between pregnant women with human immunodeficiency virus infection and HIV-negative pregnant women. However, it has been reported that in immunocompromised individuals who receive immunosuppressive therapy or individuals with hematological malignancies (2, 3, 12–15, 22–26), chronic HEV infection may develop, leading to life-threatening liver fibrosis and cirrhosis.

The poor feto-maternal outcomes in the current study may be explained by several factors, such as age structure, combination of other viral infections and comorbidities with HEV infection, late registration at the antenatal clinic and non-compliance with HEV treatment during pregnancy. It could also be attributed to non-enrolment or those who only presented for the first time at delivery date. This finding contrasts with other studies or findings that have observed uneventful foetomaternal outcomes (3, 9, 12–15, 18, 22–26). Furthermore, poor outcomes may also be attributed to socioeconomic status and culture-based practices for ensuring that certain rituals are performed before visiting a medical antenatal healthcare centre for delivery.

## Limitations

The major limitations of the present study were that it was tertiary hospital based and the sample size was small; as a result, the study did not truly reflect the actual HEV seroprevalence in Nigeria. The circulating HEV genotypes in the pregnant women were not investigated in this study. HEV IgM antibodies and viral RNA were not investigated. The COVID-19 pandemic constituted a serious hindrance during sample collection, and many pregnant women refused to enroll in the study for fear of COVID-19 and the stigma that was associated with it.

## Conclusion

This study highlighted an unexpectedly high seroprevalence of HEV and poor feto-maternal outcomes in pregnant women residing in a rural and urban setting of central Nigeria. The study showed that the inherently high HEV seropositivity and poor feto-maternal outcomes may not be attributed to HEV viral hepatitis only but may be a combination of extrinsic and intrinsic factors. The factors included late antenatal registration and non-compliance with HEV treatment among the pregnant women, genetic and genomic viral factors, and other viral infections (HCV, HBV, HAV) with hepatitis E virus. Considering that HEV in pregnant women may progress to poor feto-maternal outcomes and fulminant hepatitis, regular screening of pregnant women should constitute part of the routine screening program in the antenatal clinic. HEV screening programs should be included in national guidelines for maternal-child healthcare.

All data are available in the manuscript.

## Data availability statement

The original contributions presented in this study are included in the article/supplementary material, further inquiries can be directed to the corresponding author.

## Ethics statement

Ethical approval was obtained from the Institutional Review Board (IRB) of Federal Medical Centre, Keffi (No. KF/REC/02/21) and Uniklinikum Ethical Committee, University of Freiburg (No. 21-1233). The patients/participants provided their written informed consent to participate in this study.

## Author contributions

PE: conceptualization, designed the project, collected the data, performed the statistical analysis, and contributed to writing the manuscript. AW: supervised the entire research work, contributed to writing of manuscript and data analysis. AY: contributed to writing of the manuscript and the statistical data analysis. PM: conceptualization, original draft of manuscript, funding acquisition, and data analysis. All authors contributed to the article and approved the submitted version.
